# Consumer-Brand Relationships under the Marketing 3.0 Paradigm: A Literature Review

**DOI:** 10.3389/fpsyg.2017.00252

**Published:** 2017-02-22

**Authors:** Mónica Gómez-Suárez, María Pilar Martínez-Ruiz, Noemí Martínez-Caraballo

**Affiliations:** ^1^Finance and Marketing Department, Universidad Autónoma de MadridMadrid, Spain; ^2^Department of Business Administration, University of Castilla-La ManchaAlbacete, Spain; ^3^Economy and Management, Centro Universitario de la Defensa de ZaragozaZaragoza, Spain

**Keywords:** brand love, consumer behavior, customer engagement, marketing 3.0, values-driven era

## Abstract

Consumer-brand relationships encompass several dimensions, most of which have attracted growing research attention during the last years. Building these relationships is especially important in the marketing 3.0 era, where it is suggested that customers will choose those brands that satisfy their deepest needs. With these ideas in mind, this article provides a review of two key concepts implied in such relationships: brand love and customer engagement. Although both conceptions focus on different stages of consumer-brand relationships, they actually cover different perspectives on the same process. Moreover, they come from diverse conceptual paradigms: whilst brand love comes from the psychology discipline, engagement derives from diverse areas of the marketing field (e.g., the service-dominant logic perspective). However, their further empirical developments have taken place in marketing. Besides, both terms appear to be applied to different empirical perspectives: brand love is usually linked to the Fast Moving Consumer Goods industry and customer engagement to services.

## Introduction

Society is changing at an ever-increasing rate, especially since the beginning of the 21st century due, among other reasons, to the increasing diffusion of information and communication technologies (ICTs). These changes are producing many modifications in the consumers’ behavior, as well as in the relationships they establish with companies, derived from the huge possibilities that ICTs offer. For instance, ICTs allow sharing information about firms, theirs products and their brands at a global level, across boundaries (e.g., Parasuraman and Zinkhan, 2002; Yadav and Varadarajan, 2005). In view of this situation, it is not surprising that by the middle of the first decade of the 21st century the concept of Marketing 3.0 –as known as the “values-driven era”– has emerged. It is a type of marketing that tries to face and respond to the current challenges, derived from globalization issues (Kotler et al., 2010), among others.

In this scenario, one of the trendy research lines in marketing, the study of consumer-brand relationships, reinforces the work of Papista and Dimitriadis (2012). In particular, the emergence of Marketing 3.0 is one of the powerful reasons why this particular research line has been receiving increasing attention during the last years. Marketing 3.0 emphasizes the need to take care of customers not as mere consumers, but as complex and multi-dimensional human beings. Under this paradigm, the role of brands as identifiers of products and firms has been overcome. Companies must posit their brands instead to seek to address to social, economic, and environmental issues as a way of engaging with society (Jiménez-Zarco et al., 2014).

Two of the main concepts regarding consumer-brand relationships are: brand love and customer engagement. Hence, a comprehensive literature review of these concepts is provided in this paper. In particular, we begin by offering a brief review of the relevant literature on Marketing 3.0 and its managerial implications. Then, the concepts of brand love and customer engagement are presented. Finally, conclusions and managerial guidelines are provided.

## Conceptual Background: Brand Love and Customer Engagement as Key Concepts in the Marketing 3.0 Context

In the values-driven era, people demand to be treated not as just simple consumers; instead, they want to be treated as whole human beings with minds, hearts, and spirits (Kotler et al., 2010). Emerged as a response to the desire of people to growingly express creativity, values and spirituality, Marketing 3.0 makes companies behave as active agents, aiming at positioning themselves as companies whose brands have respect and admiration (Jiménez-Zarco et al., 2014).

This has several implications for brand management. On the one hand, in order to positioning a brand, companies must take into account that the way to differentiate the brand sometimes is not related to the mere fact of attaching the brand itself to a product or service -it should rather link the brand to a particular set of potential emotional benefits that it promises to deliver to the consumer. On the other hand, it is expected that those brands that are acknowledged as ethical elicit positive emotional responses among its consumers and invoke a stronger level of brand affect among them (Glomb et al., 2011; Martínez-Cañas et al., 2016).

Given the relevance of the affective and emotional links usually generated between brands and consumers, companies must take them into account in order to build and manage sustainable brands along time. With this regard, it is interesting to mention how two areas of research have sparked particular interest in the marketing literature because of their special links with emotions: brand love and consumer engagement (Gómez-Suárez et al., 2016).

The first empirical studies carried out to examine these intense consumer-brand relationships were those analyzing the first of these concepts. Nevertheless, Sallam (2014) outlined how it was first introduced by Shimp and Madden (1988), the managerial interest for brand love came after the publication of Roberts (2006). For this author, “lovemarks” were brands that were positioned not only in the mind but also at the heart, causing intrigue, excitement, appreciation and desire among their customers (Pawle and Cooper, 2006). Subsequently, Professor Aaron Ahuvia and his co-authors carried out several research works (e.g., Ahuvia, 1993; Carroll and Ahuvia, 2006; Batra et al., 2012; Ahuvia et al., 2014) in order to conceptualize brand love and to draw several empirical applications.

Brand love represents an intimate experience of the customers –in positive emotional terms– toward the brand. Authors who have previously conducted research on this topic provide different definitions, such as “*a product, service, or entity that inspires loyalty beyond reason*” (Pawle and Cooper, 2006, p. 39), “*the degree of passionate emotional attachment a satisfied consumer has for a particular trade name*” (Carroll and Ahuvia, 2006, p. 81) or “*a higher-order construct including multiple cognitions, emotions, and behaviors, which consumers organize into a mental prototype*” (Batra et al., 2012, p. 2). Regarding brand love, the following papers are worthy of a special mention: Fetscherin et al. (2014), Sarkar and Sreejesh (2014), Huber et al. (2015), Vernuccio et al. (2015), and Kaufmann et al. (2016).

In the management literature, (employee) engagement was first conceptualized by Kahn (1990). High customer engagement, like employee engagement, means that customers present themselves physically, cognitively, and emotionally –i.e., during the service encounter–, particularly since customers have been considered as “partial employees” or “co-producers” in some service situations (Baron et al., 1996; Bendapudi and Leone, 2003)^1^. Nowadays, several organizations consider important consumer engagement and know that it needs to be at the center of the customer service strategy.

In general terms, customer engagement is focused on the interactions between the firm and the customers and is a key research priority of the Marketing Science Institute (MSI). Focusing on customer engagement, the following papers are worthy of a special mention: Brodie et al. (2011), Hollebeek and Chen (2014), and Hollebeek et al. (2014).

According to Hollebeek (2011), there is a lack of consensus pertaining to the definition of engagement-based concepts. Among all of them, we have chosen the two most cited in the literature: customer engagement is *“a multidimensional concept comprising cognitive, emotional, and/or behavioral dimensions, [which] plays a central role in the process of relational exchange”* (Brodie et al., 2011, p. 3), and/or *“the level of the customer’s (or potential customer’s) interactions and connections with the brand or firm’s offerings or activities, often involving others in the social networks created around the brand/offering/activity”* (Vivek et al., 2014, p. 406).

Albert et al. (2008) and Batra et al. (2012) develop measurement scales of brand love, which would enable identifying both brands and product categories that might benefit from a consumer-brand relationship. In order to measure customer brand engagement (CBE), Hollebeek et al. (2014) and Vivek et al. (2014) develop and validate CBE scales in different settings.

Although the aforementioned definitions contain common elements, and three papers –Bergkvist and Bech-Larsen, 2010; Sarkar and Sreejesh, 2014; Wallace et al., 2014– have been published that partially investigate the existence of a certain connection between both concepts; when exploring which underlying theories have supported previous research, draws the conclusion that they start from different paradigms.

Basically, it could be said that there has been a fragmented interpretation depending on the research tradition in which they have been supported. Thereby, customer engagement have been developed from several marketing theories, such as the expanded domain of relationship marketing (Morgan and Hunt, 1994), or the service-dominant logic perspective (Vargo and Lusch, 2004, Vargo and Lusch, 2008); whilst brand love is based on theories from the psychology domain, such as the triangular theory of interpersonal love (Sternberg, 1986). Another important issue arises: different names are sometimes used when referring to the same concepts. For instance, the concept of self-congruity –that derives from branding theories– has nearly the same meaning as identity, derived from the identification theory.

Finally, both concepts are frequently applied to different objects of study. In general, brand love has been analyzed in research applied to consumer brands in the Fast Moving Consumer Goods industry, whilst customer engagement has been used mainly in the service sector. However, there are studies that have tried to overcome this limitation. Especially for fulfilling this research gap, in Long-Tolbert and Gammoh (2012), a model have been developed in order to apply brand love to the case of intangible goods.

## Conclusion

Consumer-brand relationships have received increasing attention during last years, being Kaufmann et al. (2016) one of the last contributions to this topic. This paper contributes to the understanding of the complex brand relationships consumers have, by exploring the dynamics between brand love and co-creation. In the literature on consumer-brand relationships, there are two concepts that determine a very intense link between them; i.e., brand love and consumer engagement. In this paper, a conceptual delimitation of these two key terms has been done.

To this end, a brief reflection has been started on the context of Marketing 3.0 and how it has boosted the research line dealing with the study of relationships between consumers and brands. Under this paradigm, consumers try to acquire those brands that allow them to especially meet their deeper needs for social, economic, and environmental justice. Namely, consumers no longer consider only the role of brands as mere identifiers of products, services or companies, but try to go further, basing their brand choices on potential associations and emotional benefits that a specific brand is susceptible to provide them. Then, a conceptual demarcation of terms brand love and customer engagement has been established. In particular, it has become clear how such terms derived from differentiated disciplines and have often been applied to different empirical situations.

Nowadays, given the profound changes taking place in markets, it is necessary to pay attention to how the consumer-brand relationships continue to evolve. In short, among future trends, there should be considered those that are likely to have a greater impact on these relationships, such as the opportunities offered by an efficient management of Big Data and the advent of Marketing 4.0. Big data helps companies to build strong relationships. Marketing 4.0 is the marketing of big data (Jiménez-Zarco et al., 2017). Marketing 4.0, from human-centric to content marketing, helps companies to adapt to the changing nature of customer paths in the digital economy (Kotler et al., 2017). Marketing 4.0 requires: firstly, a depth knowledge about the evolution of marketing, especially about Marketing 3.0, and secondly, an analysis of how technology –not only the Internet and social media– can be used to design marketing strategies that enhance the brand-consumer relationships.

## Author Contributions

All authors listed, have made substantial, direct, and intellectual contribution to the work, and approved it for publication.

## Conflict of Interest Statement

The authors declare that the research was conducted in the absence of any commercial or financial relationships that could be construed as a potential conflict of interest.

## Footnotes

1 Co-creation is “the joint creation of value by the company and the customer; allowing the customer to co-construct the service experience to suit their context” (Prahalad and Ramaswamy, 2004, p. 8).
